# Pulmonary function trajectories in COVID-19 survivors with and without pre-existing respiratory disease

**DOI:** 10.1038/s41598-024-67314-0

**Published:** 2024-07-17

**Authors:** Debbie Gach, Rosanne J. H. C. G. Beijers, Roel van Zeeland, Vivian van Kampen-van den Boogaart, Rein Posthuma, Annemie M. W. J. Schols, Joop P. van den Bergh, Frits H. M. van Osch

**Affiliations:** 1 Department of Respiratory Medicine, NUTRIM, Institute of Nutrition and Translational Research in Metabolism, Maastricht University Medical Centre+, Maastricht, The Netherlands; 2 Department of Clinical Epidemiology, VieCuri Medical Centre, Venlo, The Netherlands; 3 Department of Respiratory Medicine, VieCuri Medical Centre, Venlo, The Netherlands; 4 Department of Research and Development, Ciro+, Hornerheide 1, 6085 NM Horn, The Netherlands; 5 Department of Internal Medicine, VieCuri Medical Centre, Venlo, The Netherlands; 6 Department of Internal Medicine, NUTRIM, Institute of Nutrition and Translational Research in Metabolism, Maastricht University Medical Centre+, Maastricht, The Netherlands; 7 Department of Epidemiology, GROW, Research Institute for Oncology and Reproduction, Maastricht University Medical Centre+, Maastricht, The Netherlands

**Keywords:** Respiratory tract diseases, Epidemiology

## Abstract

A significant proportion of COVID-19 survivors still experience a reduced diffusion capacity three and twelve months after discharge. We aimed to compare pulmonary function trajectories between hospitalized COVID-19 patients with pre-existing respiratory disease (PRD) and patients without pre-existing respiratory disease (Non-PRD) at three and twelve months after hospital discharge. This single-centre retrospective cohort study included COVID-19 patients admitted to the VieCuri Medical Centre (Venlo, the Netherlands) between February and December 2020 that were invited to the outpatient clinic at three and twelve months after discharge. During this visit, pulmonary function tests were performed and impairments were based on lower limit of normal. Data of 239 patients were analysed (65% male, 66 ± 10 years, and 26% with a history of respiratory disease). Three months after discharge, 49% and 64% of the Non-PRD patients (n = 177) and PRD patients (n = 62) had a low diffusion capacity, respectively. This improved over time in Non-PRD patients (*p* = 0.003), but not in PRD patients (*p* = 0.250). A low diffusion capacity was still observed in 34% and 57% of the Non-PRD and PRD group, respectively, twelve months after discharge. Pulmonary function impairments, mainly a reduced diffusion capacity, are observed among hospitalized COVID-19 patients with PRD and Non-PRD, at three and twelve months follow-up. Although diffusion capacity impairments restore over time in Non-PRD patients, poor recovery was observed among PRD patients.

## Introduction

Since the onset of the COVID-19 pandemic in December 2019, 772 million confirmed COVID-19 cases have been reported worldwide according to the World Health Organization (WHO)^1^. Within the Netherlands, 6.6 million cases have been confirmed until March 2023, from which around 9% of the people required hospitalization during the acute COVID-19 phase, thereby placing an immense burden on hospitals and health care systems^2^. Hospitalized COVID-19 patients most commonly presented symptoms such as fever, fatigue, cough, and dyspnoea, leading to the need of oxygen therapies and sometimes even admission to intensive care units (ICUs) during hospitalization^3,4^.

While part of the patients recovers during the acute phase (within four weeks), half of the hospitalized COVID-19 patients still experience symptoms three months after the acute infection^5^. This feature of ongoing symptoms is often defined as long COVID or Post COVID-19 condition^5^. The most common persisting symptoms reported by COVID-19 survivors three months after discharge include fatigue, dyspnoea, and sleep- and memory problems^5^. Even one year after infection, one-third of the hospitalized COVID-19 patients is not feeling fully recovered and experiences symptoms such as fatigue, muscle weakness, poor sleep, memory- and concentration problems, and breathlessness^6,7^, which largely impacts the health-related quality of life of these patients^8^.

Some symptoms associated with the post COVID-19 condition have been found to be related to impairments in pulmonary function^8,9^. Long-term pulmonary function impairments mainly include a reduced diffusion capacity, which has been reported in up to 52–56% of the hospitalized COVID-19 patients three months after discharge^10–12^. A large prospective cohort study shows that even one year after discharge, persistent pulmonary function damage, i.e. abnormal diffusion capacity and declined total lung capacity (TLC), are reported in 39% and 42% of the COVID-19 survivors, respectively^13^. Although in the majority of the patients, these pulmonary function impairments improve over time, still one-third of the COVID-19 patients that were hospitalized during the acute phase of the infection have a reduced diffusion capacity one year later^14^. Acute disease severity, female gender, and the presence of co-morbidities including chronic respiratory disease, diabetes, and hypertension were associated with worse long-term pulmonary function outcomes in hospitalized COVID-19 patients^10,13,14^. Next to the changes in static lung volumes and gas exchange function, impairments in respiratory muscle function have been reported among hospitalized and non-hospitalized COVID-19 patients^15–17^. More specifically, a large retrospective study has revealed significant reductions in inspiratory muscle strength in non-hospitalized COVID-19 patients at three months follow-up^15^. Hereby, they also found a strong association between a decreased inspiratory muscle strength and a higher dyspnoea sensation^15^. Accordingly, in a cohort of hospitalized COVID-19 patients, 88% of the survivors experienced a reduced inspiratory muscle strength approximately five months after the acute infection^16^. These findings indicate respiratory muscle dysfunction in post COVID-19 patients, which may be attributed to impairments in respiratory muscle contractibility^18^.

Especially in patients with already compromised respiratory muscle contractibility and decreased pulmonary function, as in chronic obstructive pulmonary disease (COPD) and asthma, it is important to monitor long-term changes in pulmonary function since they might be more susceptible for additional pulmonary impairments after a SARS-CoV-2 infection^18,19^. However, current data is lacking on the long-term pulmonary function changes in a large cohort of hospitalized COVID-19 patients that distinguishes between patients with- and without pre-existing respiratory disease. The aim of this study is therefore to compare pulmonary function trajectories between hospitalized COVID-19 patients with pre-existing respiratory disease (PRD) and patients without pre-existing respiratory disease (Non-PRD) at three and twelve months after hospital discharge.

## Methods

### Study design and population

This single-centre retrospective cohort study included all COVID-19 patients admitted to the VieCuri Medical Centre in Venlo, the Netherlands, in the first (between February and June 2020) and second wave (between July and December 2020) of the COVID-19 pandemic. Patients were eligible for inclusion in this study if they were hospitalized with a confirmed SARS-CoV-2 infection based on positive reverse transcription polymerase chain reaction, visited the post COVID-19 aftercare program three and twelve months after hospital discharge, and were aged above 18 years.

After hospital discharge, all patients were invited at the multidisciplinary COVID-19 aftercare program for evaluation by the departments of Internal Medicine and Pulmonology at three months follow-up. The aftercare program included amongst others of a pulmonary function test. Based on the clinical outcomes retrieved during the outpatient clinic visit at three months, patients were referred to a specialist if necessary. A standardized follow-up visit at twelve months was planned for patients that experienced symptoms at three months follow-up, while the twelve months follow-up visit was facultative for patients who did not experience symptoms anymore. In case patients did not visit the outpatient clinic, reasons for not visiting were retrieved from the electronic medical records.

According to the medical ethics committee of Maastricht University Medical Centre+, the current study is beyond the scope of the Medical Research Involving Human Subjects Act (WMO) (2021–3059). Informed consent for all patients was waived due to the retrospective approach of the study and the exceptional circumstances related to the COVID-19 crisis in accordance with the national guidelines and European privacy law.

### Data collection

Baseline and hospitalization characteristics were collected during the acute COVID-19 phase defined as the time between hospital admission and discharge. Pulmonary function tests were performed during the outpatient clinic visits at three and twelve months follow-up. A detailed description of data collection and pulmonary function assessment procedures are provided in the supplemental material (see Supplementary 1).

### Statistical analysis

For comparison of patient characteristics and pulmonary function outcomes between the PRD and Non-PRD group, a Chi-Square test was calculated and an independent samples T-test or Mann–Whitney U-test as appropriate. To compare pulmonary function outcomes between three and twelve months, a paired samples T-test or a Wilcoxon sign rank test was used as appropriate. A McNemar test was performed to assess significant changes over time for dichotomous variables (under/above lower limit of normal (LLN)). Analyses were performed using IBM SPSS statistics, version 28. A *p-*value < 0.05 was considered statistically significant.

## Results

### Patient characteristics

**Figure 1:**
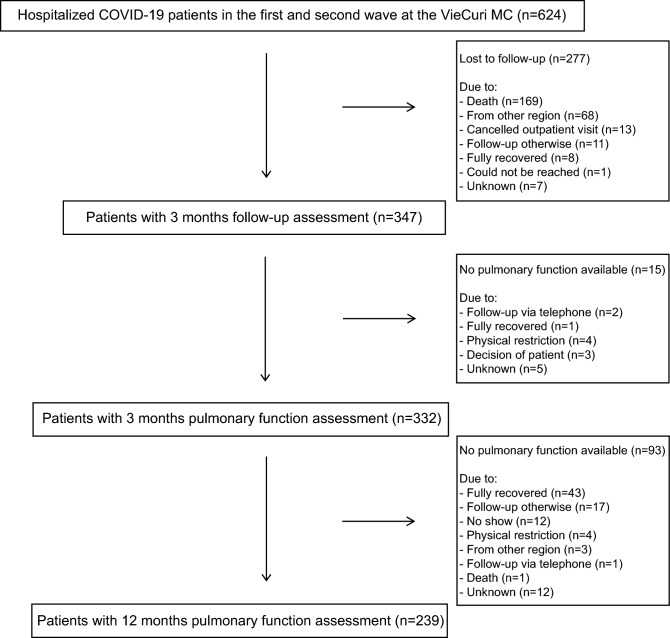
Flowchart of the (included) study population.

In total, 624 patients were hospitalized during the first and second wave at the VieCuri Medical Centre, of which 169 patients died before the 3 months follow-up assessment (see Fig. 1). Eventually, 347 out of the 387 patients (90%) who survived and could have attended the outpatient clinic had a three months follow-up assessment, with 332 patients (96%) also completing the pulmonary function assessment. Ninety-three patients (28%) did not complete the pulmonary function assessment at twelve months with the main reason that they felt fully recovered. Consequently, 239 patients had a pulmonary function assessment at three and twelve months, and were included for analyses in this study.

The mean age of the study group was 66 years with the majority being male (65%) of whom 62 (26%) had PRD (see Table 1). The most frequently reported co-morbidities were hypertension (47%), obesity (31%), and diabetes (31%). Median length of hospital stay was 7 days (4–14) and 20% of the patients had been admitted to the ICU with a median stay of 12 days (5–34). Time between discharge and 3 and 12 months follow-up was 110 days (96–132) and 384 days (353–422), respectively. Of the 239 patients, 213 (89%) received nasal oxygen therapy during hospitalization. No differences were observed in patient and hospitalization characteristics between PRD and Non-PRD patients. Baseline characteristics of the 93 excluded patients due to lacking the twelve months visit compared to the 239 included patients with a complete follow-up are provided in the supplemental material (see Supplementary 2; Table S1).

**Table 1:** Patient characteristics of the hospitalized COVID-19 patients with both 3 and 12 months pulmonary function assessment for the total group and stratified by PRD.

Patient characteristics	Total (n = 239)	PRD patients (n = 62)	Non-PRD patients (n = 177)	*p*-value
Waves				
*First*	159 (67)	39 (63)	120 (68)	0.482
*Second*	80 (33)	23 (37)	57 (32)	
Age in years	66 ± 10	68 ± 10	66 ± 10	0.199
Male	155 (65)	42 (68)	113 (64)	0.580
BMI in kg/m^2^	28 ± 4.7	28 ± 3.9	29 ± 5.0	0.481
BMI categories				0.596
*Normal weight* (18.5–24.9 kg/m^2^)	53 (22)	15 (24)	38 (22)	
*Overweight* (25.0–29.9 kg/m^2^)	110 (47)	31 (50)	79 (45)	
*Obese* (≥ 30.0 kg/m^2^)	73 (31)	16 (26)	57 (33)	
**Co-morbidities**				
Hypertension	112 (47)	32 (52)	80 (45)	0.635
Type 2 diabetes	73 (31)	21 (34)	52 (29)	0.565
Obesity	73 (31)	16 (26)	57 (33)	0.428
Chronic cardiac disease	56 (23)	15 (24)	41 (23)	0.937
Chronic respiratory disease	62 (26)	–	–	*–*
Chronic kidney disease	24 (10)	6 (10)	18 (10)	0.992
Chronic neurologic disease	22 (9)	8 (13)	14 (8)	0.403
Rheumatologic disorder	29 (12)	7 (11)	22 (12)	0.936
Autoimmune disorder	27 (11)	6 (10)	21 (12)	0.525
Malignant neoplasma	21 (9)	7 (11)	14 (8)	0.681
CCI score^a^	3 (2–4)	4 (2–5)	3 (2–4)	–
**Hospital stay**				
Days from onset to admission^a^	8 (7–12)	8 (6–13)	8 (7–12)	0.825
Days from admission to discharge^a^	7 (4–14)	7 (4–13)	7 (5–14)	0.736
ICU admission	48 (20)	10 (16)	38 (22)	0.366
Length of ICU stay in days^a^	12 (5–34)	8 (4–26)	13 (9–37)	0.098
Days from discharge to three months FU^a^	110 (96–132)	104 (94–123)	111 (96–134)	0.096
Days from discharge to 12 months FU^a^	384 (353–422)	384 (360–416)	384 (352–426)	0.985
**Oxygen treatments during hospital stay**				
Nasal oxygen therapy	213 (89)	53 (86)	160 (90)	0.285
Non-invasive ventilation	10 (4)	2 (3)	8 (5)	0.785
Invasive ventilation	39 (16)	6 (10)	33 (19)	0.067

Data are shown as median ± SD or n (%) unless indicated otherwise.

^a^Median (IQR). Abbreviations: BMI, body mass index; CCI, charlson co-morbidity index; FU, follow-up; ICU, intensive care unit; PRD, pre-existing respiratory disease.

### Pulmonary function outcomes at three months

**Figure 2:**
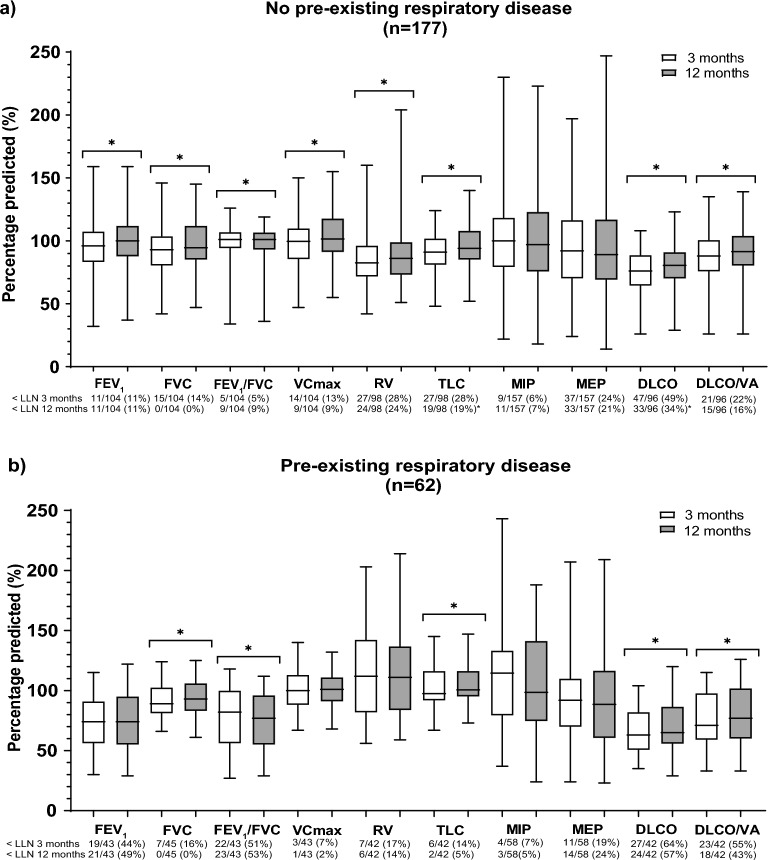
(**a**), (**b**) Pulmonary function of the hospitalized COVID-19 patients with both 3 and 12 months pulmonary function assessment and stratified by PRD. Data are shown as median (IQR) for continuous variables and n (%) for categorical variables. ^*^Indicates a significant difference between 3 and 12 months follow-up,* p* < 0.05. Abbreviations: DLCO, diffusing capacity of the lungs for carbon monoxide; FEV_1_, forced expiratory volume in 1 s; FVC, forced vital capacity; MEP, maximum expiratory pressure; MIP, maximum inspiratory pressure; RV, residual volume; TLC, total lung capacity; VA, alveolar volume; VCmax, maximum vital capacity.

In the Non-PRD group, percentage predicted for diffusion capacity of the lungs for carbon monoxide (DLCO) was lowest (76% (64–89); see Fig. 2a). Median DLCO was also lowest in the PRD group (63% (51–82); see Fig. 2b), and this was lower than the Non-PRD group (*p* = 0.003). Most prevalent pulmonary function impairments were found for DLCO (49%), TLC (28%), RV (28%), and MEP (24%) in Non-PRD patients (see Fig. 2a). In PRD patients, 64%, 14%, 17%, and 19% showed an impaired DLCO, TLC, RV, and MEP (see Fig. 2b), respectively, which was not different compared to the Non-PRD patients (*p* > 0.05).

### Pulmonary function changes between three and twelve months

**Figure 3:**
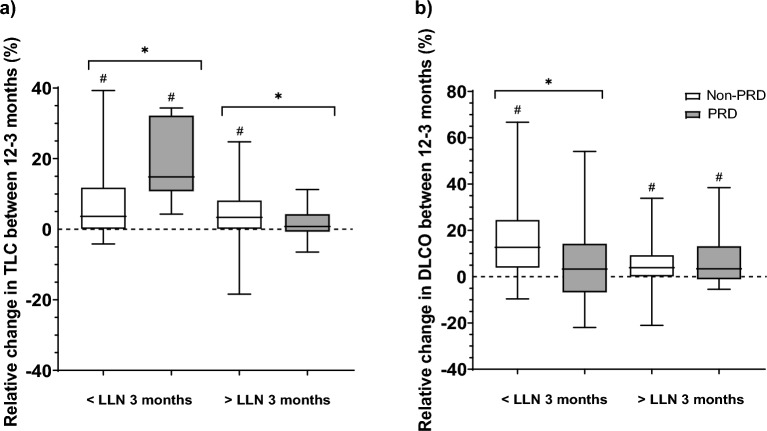
(**a**), (**b**) Pulmonary function changes in TLC (**a**) and DLCO (**b**) of the hospitalized COVID-19 patients with both 3 and 12 months pulmonary function assessment and stratified by PRD as well as below/above LLN at 3 months follow-up. Data are shown as median (IQR). ^*^Indicates a significant difference between the PRD and Non-PRD group,* p* < 0.05. ^#^Indicates a significant difference between 3 and 12 months follow-up,* p* < 0.05. Abbreviations: DLCO, diffusing capacity of the lungs for carbon monoxide; LLN, lower limit of normal; PRD, pre-existing respiratory disease; TLC, total lung capacity.

Overall, percentage predicted DLCO increased over time in the Non-PRD group (*p* < 0.001), but still remained the most impaired pulmonary function variable at twelve months (81% (70–91); see Fig. 2a). In the PRD group, median DLCO also increased over time to 65% (56–87) at twelve months (*p* = 0.008; see Fig. 2b), but was still lower than the Non-PRD group (*p* = 0.002). Over time, prevalence of DLCO and TLC impairments decreased to 34% and 19% in Non-PRD patients at twelve months, respectively (*p* < 0.05; see Fig. 2a). However, RV and MEP abnormalities were still observed in 24% and 21% at twelve months, respectively (*p* > 0.05). In patients with PRD, prevalence of DLCO, TLC, RV, and MEP impairments were still present in 57%, 5%, 14%, and 24% at twelve months, respectively (*p* > 0.05; see Fig. 2b). DLCO impairments were higher in the PRD group compared to the Non-PRD group at twelve months (*p* = 0.012), while the prevalence of TLC abnormalities were more frequently seen in Non-PRD patients than PRD patients (*p* = 0.025). Figure 3 shows specific analyses within the groups that had either impaired TLC or DLCO at three months. In PRD and Non-PRD patients with an impaired TLC at three months, increments over time were seen in TLC of 15% (11–32) and 4% (0–12) (*p* = 0.009 and *p* < 0.001, respectively; see Fig. 3a). The increment in TLC was larger in the PRD group than the Non-PRD group (*p* = 0.034). In patients with a normal TLC at three months, no difference over time was observed in TLC in the PRD group (1% (− 1–4); *p* = 0.108), while a small increase of TLC was observed in the Non-PRD group (3% (0–8); *p* < 0.001).

An increase in DLCO of 13% (4–25) was seen in Non-PRD patients with an abnormal DLCO at three months (*p* < 0.001; see Fig. 3b). This was higher compared to the PRD patients with an impaired DLCO at three months (*p* = 0.032), in which only small increments over time were observed (3% (− 7–14); *p* = 0.088). Patients with a normal DLCO at three months showed increments over time in DLCO in the PRD and Non-PRD group (3% (− 1–13); *p* = 0.041 and 4% (0–9); *p* = 0.006, respectively).

## Discussion

This study shows that pulmonary function abnormalities, mainly a reduced DLCO, are seen in two-third and half of the hospitalized COVID-19 patients with PRD and Non-PRD, respectively, three months after discharge. Over time, DLCO impairments decreased to one third in the Non-PRD group, whereas no changes were observed in the PRD group with still two-third of the patients experiencing a reduced DLCO twelve months after discharge.

Results of the current study confirm previous research showing that DLCO abnormalities are the most frequently observed long-term pulmonary function impairment among hospitalized COVID-19 patients^8^. Although DLCO impairments mostly restore over time in Non-PRD patients^14,20,21^, our cohort of PRD patients did not show improvements in DLCO abnormalities over time. These patients might already have an impaired DLCO caused by the pathological characteristics of their underlying disease including emphysema and pulmonary hypertension, which are known to negatively influence the diffusion capacity of the lungs^22,23^. Accordingly, in PRD patients with an abnormal DLCO at three months, only a small increase in DLCO (3%) was seen over time, while a larger improvement (13%) was noticed among Non-PRD patients. The exact pathophysiological mechanism behind these pulmonary function impairments post COVID-19 remains to be elucidated but prolonged systemic inflammation and (pulmonary) vascular damage have been proposed^24,25^. Furthermore, protracted inflammation caused by the SARS-CoV-2 infection along with the already pro-inflammatory state of the lungs as seen in COPD could potentially explain the poor recovery in PRD patients^23^.

Reduced lung volumes are also frequently reported among post COVID-19 patients^8^. Accordingly, in our cohort of Non-PRD patients, 28% demonstrated a reduced TLC and RV three months after discharge, which was still present in 19% and 24%, respectively, twelve months after discharge, thereby indicating a restrictive pattern in COVID-19 survivors similar to other corona-virus induced-syndromes^26^. In contrast, a reduced TLC (5%) was less present in PRD patients at twelve months follow-up. This could be explained due to the fact that in obstructive pulmonary diseases such as COPD, increments in TLC are usually observed due to the loss of elastic recoil in the lungs, thereby reflecting hyperinflation, which subsequently results in less patients falling below LLN in the PRD group^27^.

Respiratory muscle dysfunction might play a key role in the changed lung volumes among COVID-19 survivors. Several mechanisms have been hypothesized via which a SARS-CoV-2 infection may affect respiratory muscle performance such as by reducing respiratory muscle contractibility and by paralysis of the unilateral diaphragm^28,29^. We found that an impaired MEP was still noted in 24% and 21% of the PRD and Non-PRD patients at twelve months, respectively, which was not improved over time in both groups. Respiratory muscle weakness is also a main feature among patients with COPD and this is known to contribute to the high levels of dyspnoea in these patients^30,31^. Since it has become apparent that COVID-19 may cause damage to the respiratory muscles, this has also been proposed as one of the main factors leading to prolonged respiratory complications in post COVID-19 patients^28^.

The poor recovery in pulmonary function impairments, especially in DLCO, is concerning in PRD patients, since it is known that DLCO impairments are associated with an increased all-cause mortality risk and decreased health-related quality of life in patients with COPD^32,33^. Zhang et al.^34^ was the first study to describe lung function trajectories in post COVID-19 patients up to 2 years after infection. They showed improvements in pulmonary function outcomes during the first year after infection, as in line with our results in Non-PRD patients, but an alarming declining trend was found during the second year^34^. This highlights the urgent need for longer-term follow-up measurements beyond one year to monitor the exact trajectory of pulmonary function patterns in post COVID-19 patients.

This study has several limitations. First, the lack of data on pulmonary function outcomes before the SARS-CoV-2 infection makes it difficult to establish any causality. Secondly, we only included patients with three and twelve months follow-up data which has led to some overestimation of the pulmonary function impairments, since DLCO abnormalities were less present in PRD and non-PRD patients with only 3 months follow-up data (52% and 35%, respectively). Although 54% of the patients being excluded due to the lack of a twelve months visit reported other reasons than being fully recovered, potentially limiting the overestimation of respiratory impairment at twelve months, selection bias cannot be avoided. Also, the lack of chest CT-scan data limits the ability to explore the full spectrum of pulmonary outcomes in COVID-19 survivors. Lastly, this study only selected hospitalized patients in the first and second wave of the pandemic, which makes the generalizability of our results less for the latter waves of the pandemic and for non-hospitalized post COVID-19 patients. However, the results give a reliable representation of the long-term pulmonary function outcomes in COVID-19 survivors from both general ward and ICU using real-world data from clinical practice, with the unique aspect of distinguishing between PRD and Non-PRD patients. This gave us the opportunity to acknowledge a highly vulnerable patient population.

In conclusion, this study shows that pulmonary function impairments, mainly a reduced DLCO, are observed in PRD and Non-PRD hospitalized COVID-19 patients at three and twelve months follow-up. Although DLCO impairments restore over time in Non-PRD patients, poor recovery was noted among PRD patients. Future longitudinal studies with longer follow-up periods beyond one year are needed to unveil the precise pattern of respiratory complications in post COVID-19 patients with- and without PRD, which may subsequently contribute to personalized disease management in the future.

### Supplementary Information

Supplementary Information.

## Data Availability

Datasets and scripts used in this study are available from the corresponding author upon request.
