# Supplementary material for: Pulmonary function trajectories in COVID-19 survivors with and without pre-existing respiratory disease
Source: Sci Rep. 2024 Jul 17;14:16571. doi: 10.1038/s41598-024-67314-0 (PMC11255309; doi:10.1038/s41598-024-67314-0)
Supplement: Supplementary file 1 — Supplementary Information. [file 41598_2024_67314_MOESM1_ESM.docx]

**Supplemental material**

**Supplementary 1**

*Data collection*

Baseline and hospitalization characteristics

Baseline and hospitalization characteristics were collected during the acute COVID-19 phase which was defined as the time between hospital admission and discharge. The following baseline characteristics were retrieved from the electronic medical records of all patients: age, gender, body mass index (BMI), and the presence of co-morbidities. Hospitalization characteristics such as length of hospital stay, type of admitted ward (ICU or nursing ward), and treatments (oxygen therapy, non-invasive and/or invasive ventilation) received during hospitalization were also retrieved from the medical records. PRD was defined as the presence of COPD and/or asthma, which was based on patient’s medical history. In addition, the Charlson Comorbidity Index (CCI) was calculated for each patient [1].

Pulmonary function assessment

Pulmonary function tests were performed during the outpatient clinic visits at three and twelve months after discharge, according to European Respiratory Society guidelines [2] on a MasterScreen^TM^ Body and MasterScreen^TM^ PFT (Pangas, Dagmersellen) by use of the SentrySuite V3.0.5. Software. Pre-bronchodilator spirometry was performed to determine forced expiratory volume in one second (FEV_1_) and forced vital capacity (FVC). Body plethysmography was conducted to assess total lung capacity (TLC), residual volume (RV), and maximum vital capacity (VCmax). As an indicator for respiratory muscle strength, maximal inspiratory- and expiratory pressure (MIP and MEP) were measured. The single-breath method was used to measure diffusion capacity of the lungs for carbon monoxide (DLCO). Additionally, DLCO per unit alveolar volume was calculated (DLCO/VA). Pulmonary function outcomes were reported as percentage of predicted values [3]. Lower limit of normal (LLN), defined as the 5th percentile according to the standardized multi-ethnic reference values for spirometry from the Global Pulmonary function initiative, was used to report pulmonary function impairments [4, 5].

**Supplementary 2**

*Patient characteristics subgroup*

Patients with both three and twelve months pulmonary function outcomes were significantly more hospitalized during the first wave and were more often males as compared to the group with only three months pulmonary function outcomes (159 (67%) vs 44 (47%); *p*=0.001 and 155 (65%) vs 46 (50%); *p*=0.010, respectively) (see Table S1). In addition, a significant longer time between symptom onset and admission (8 days (7-12) vs 7 days (5-11); *p*=0.014, respectively), and admission and discharge (7 days (4-14) vs 5 days (3-9); *p*=0.003, respectively), as well as a significant higher amount of the use of invasive ventilation (39 (16%) vs 4 (4%); *p*=0.011) was seen among patients with both three and twelve months follow-up data compared to patients with only three months follow-up data (see Table S1).

**Table S1: Patient characteristics of the hospitalized COVID-19 patients with only three months pulmonary function assessment and with both three and twelve months pulmonary function assessment.**

| **Patient characteristics** | **Patients with only three months pulmonary function assessment**  **(n=93)** | **Patients with both three and twelve months pulmonary function assessment**  **(n=239)** | ***p*-value** | |
| --- | --- | --- | --- | --- |
| Waves  *First*  *Second* | 44 (47)  49 (53) | 159 (67)  80 (33) | **0.001** |  |
| Age in years | 69±14 | 66±10 | 0.058 |  |
| Male | 46 (50) | 155 (65) | **0.010** |  |
| BMI in kg/m^2^ | 29±4.4 | 28±4.7 | 0.676 |  |
| BMI categories  *Normal weight (18.5-24.9 kg/m²)*  *Overweight (25.0-29.9 kg/m²)*  *Obese (≥ 30.0 kg/m²)* | 22 (24)  35 (39)  34 (37) | 53 (22)  110 (47)  73 (31) | 0.387 |  |
| **Co-morbidities** |  |  |  |  |
| Hypertension | 43 (46) | 112 (47) | 0.984 |  |
| Diabetes | 19 (20) | 74 (31) | 0.055 |  |
| Obesity | 34 (37) | 73 (31) | 0.842 |  |
| Chronic cardiac disease | 24 (26) | 56 (23) | 0.847 |  |
| Chronic respiratory disease | 19 (20) | 62 (26) | 0.294 |  |
| Chronic kidney disease | 16 (17) | 24 (10) | 0.192 |  |
| Chronic neurologic disease | 11 (12) | 22 (9) | 0.754 |  |
| Rheumatologic disorder | 8 (9) | 29 (12) | 0.627 |  |
| Autoimmune disorder | 9 (10) | 27 (11) | 0.900 |  |
| Malignant neoplasma | 5 (5) | 21 (9) | 0.336 |  |
| CCI score^a^ | 4 (2 – 5) | 3 (2 – 4) | 0.148 |  |
| **Hospital stay** |  |  |  |  |
| Days from onset to admission^a^ | 7 (5-11) | 8 (7-12) | **0.014** |  |
| Days from admission to discharge^a^ | 5 (3-9) | 7 (4-14) | **0.003** |  |
| ICU admission | 13 (14) | 48 (20) | 0.197 |  |
| Length of ICU stay in days^a^ | 6 (4-15) | 12 (5-34) | 0.070 |  |
| Days from discharge to three  months FU^a^ | 105 (93-134) | 110 (96-132) | 0.489 |  |
| Days from discharge to twelve months FU^a^ | - | 384 (353-422) | *-* |  |
| **Oxygen treatments during hospital stay** |  |  |  |  |
| Nasal oxygen therapy | 75 (81) | 213 (89) | 0.051 |  |
| Non-invasive ventilation | 6 (7) | 10 (4) | 0.218 |  |
| Invasive ventilation | 4 (4) | 39 (16) | **0.011** |  |

**Table S1 legend:** Data are shown as median±SD or n (%) unless indicated otherwise. ^a^Median (IQR). Abbreviations: BMI, body mass index; CCI, charlson co-morbidity index; FU, follow-up; ICU, intensive care unit; PRD, pre-existing respiratory disease.

**References**

[1] Charlson, M. E., Pompei, P., Ales, K. L. & MacKenzie, C. R. A new method of classifying prognostic comorbidity in longitudinal studies: development and validation. *J Chronic Dis.* **40**(5), 373-83 (1987).

[2] Miller, M. R. *et al.* General considerations for lung function testing. *Eur Respir J.* **26**(1), 153-61 (2005).

[3] Hall, G. L. & Stanojevic, S. Executive GLIN, Members of the GLINE. The Global Lung Function Initiative (GLI) Network ERS Clinical Research Collaboration: how international collaboration can shape clinical practice. *Eur Respir J.* **53**(2), 1802277; 10.1183/13993003.02277-2018 (2019).

[4] Quanjer, P. H. *et al.* Multi-ethnic reference values for spirometry for the 3-95-yr age range: the global lung function 2012 equations. *Eur Respir J.* **40**(6), 1324-43 (2012).

[5] Stanojevic, S. *et al.* Official ERS technical standards: Global Lung Function Initiative reference values for the carbon monoxide transfer factor for Caucasians. *Eur Respir J.* **50**(3), 1700010; 10.1183/13993003.00010-2017 (2017).
